# Organic phosphate but not inorganic phosphate regulates *Fgf23* expression through MAPK and TGF-ꞵ signaling

**DOI:** 10.1016/j.isci.2024.109625

**Published:** 2024-03-29

**Authors:** Danielle M.A. Ratsma, Max Muller, Marijke Koedam, Johannes P.T.M. van Leeuwen, M. Carola Zillikens, Bram C.J. van der Eerden

**Affiliations:** 1 Laboratory for Calcium and Bone Metabolism and Erasmus MC Bone Centre, Department of Internal Medicine, Erasmus MC, Erasmus University Medical Center, Rotterdam, the Netherlands

**Keywords:** molecular biology, cell biology, Transcriptomics

## Abstract

One of the main regulators of phosphate homeostasis is fibroblast growth factor 23 (FGF23), secreted by osteocytes. The effects of organic versus inorganic dietary phosphate on this homeostasis are unclear. This study used MC3T3-E1 FGF23-producing cells to examine the transcriptomic responses to these phosphates. Most importantly, the expression and secretion of FGF23 were only increased in response to organic phosphate. Gene ontology terms related to a response to environmental change were only enriched in cells treated with organic phosphate while cells treated with inorganic phosphate were enriched for terms associated with regulation of cellular phosphate metabolism. Inhibition of MAPK signaling diminished the response of *Fgf23* to organic phosphate, suggesting it activates FGF23. TGF-β signaling inhibition increased *Fgf23* expression after the addition of organic phosphate, while the negative TGF-β regulator *Skil* decreased this response. In summary, the observed differential response of FGF23-producing to phosphate types may have consequences for phosphate homeostasis.

## Highlights

• Regulation of FGF23 can be studied in differentiated MC3T3-E1 osteocyte-like cells
• Only organic phosphates increase the expression and secretion of FGF23
• MAPK signaling stimulates FGF23 expression while TGF-β inhibits this
• TGF-β regulator *Skil* may prevent the regulation of FGF23 by inorganic phosphate

## Graphical abstract

**Figure:**
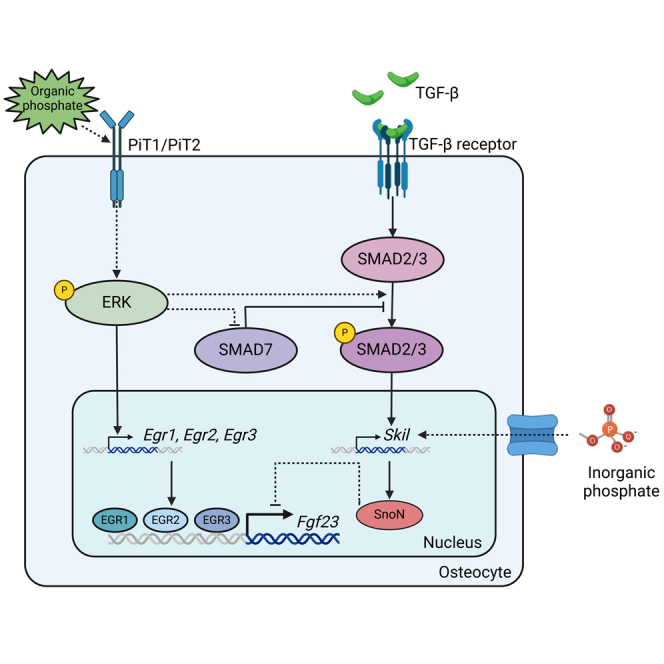

## Introduction

Phosphate is an essential nutrient for skeletal health and plays a role in numerous metabolic processes.^1^ Phosphate is present in most food sources either as naturally occurring organic phosphate or as added inorganic phosphate. In healthy people, 40–60% of the organically bound phosphate will be absorbed in the intestine. In contrast, up to 100% of the ingested inorganic phosphate can be absorbed.^2^^,^^3^ Higher dietary phosphate content will result in higher urinary and fecal phosphate excretion. Although this may seem straightforward, the dietary phosphate type could influence phosphate homeostasis by affecting its absorption and excretion.

As the Western diet drastically changed throughout the years, we now ingest more inorganic phosphate than previous generations did.^4^ In order to consume food and beverages longer after their production, food additives are frequently being added as preservatives. Phosphorus is the main component of these additives, often as phosphoric acids, orthophosphates, or polyphosphates. There is currently no recommended limit for the amount of food additives that can be used in a product.^5^ Both European law and the Food and Drug Administration (FDA) dictate that manufacturers must report which additives are used in their products, but it does not require reporting the quantities of those additives. Moreover, there are no guidelines yet for labeling of food products for phosphate, as opposed to sodium, potassium, and other salts.^2^ Since the use of food additives is widespread, inorganic phosphate from food additives can accumulate to 30% of the total phosphate intake. The high absorbability of these phosphate in the intestine will increase the phosphate burden even more.^6^

In the small intestine, both inorganic and organic phosphate from the diet are absorbed through a combination of passive (paracellular) and active (transcellular) transport mechanisms.^7^ Transcellular transport is considered dominant at low luminal phosphate concentrations, where carrier proteins actively facilitate the absorption of inorganic and organic phosphate into intestinal epithelial cells.^8^ However, with increased phosphate concentrations, as seen in a high-phosphate diet, paracellular phosphate transport becomes more prominent. In this context, passive movement of phosphate ions across the intestinal epithelium plays a dominant role. High levels of dietary inorganic phosphate, through passive transport, could contribute to elevated blood phosphate levels, potentially disrupting the balance between bound and unbound phosphate in the circulation, resulting in higher serum phosphate levels.^2^^,^^9^^,^^10^

Several studies have shown that high inorganic dietary phosphate intake and/or high normal serum phosphate levels could pose serious health risks.^2^^,^^11^^,^^12^^,^^13^^,^^14^ For example, in mice, a high inorganic phosphate diet has been associated with skeletal muscle atrophy, kidney injury, abnormal brain growth, increased lung cancer progression, and weight gain.^15^^,^^16^^,^^17^^,^^18^^,^^19^^,^^20^^,^^21^ Other studies also considered the source of dietary phosphate. Kawamura et al. (2018) found that a high inorganic phosphate containing meal resulted in a reduction of endothelium-dependent vasodilation and contributed to the development of cardiovascular disease, while a diet rich in organic phosphate had no such effect.^22^ Moreover, a study in cats showed that the source of phosphate influences the availability, serum levels and renal excretion of phosphate.^23^ Similarly, a study in dogs showed that feeding inorganic food increased serum phosphate, while organic food sources did not.^24^ In patients with kidney disease who are unable to excrete phosphate, high serum phosphate levels are associated with morbidity and mortality.^25^ But also in a healthy population high-normal serum phosphate levels have been associated with coronary calcification and cardiovascular events, with a stronger effect in men.^26^^,^^27^ Together, these findings in animal studies indicate that the source of phosphate affects the response to it leading to higher serum levels and suggest that a high inorganic phosphate load is damaging to health. It is currently unknown whether a high inorganic diet shifts the ratio of circulating inorganic and organic phosphate toward inorganic phosphate.

Maintenance of phosphate homeostasis is hormonally regulated by a kidney-bone-intestine axis.^28^ Osteocytes residing in the mineralized bone secrete fibroblast growth factor 23 (FGF23) in response to rising serum phosphate levels.^29^ FGF23 binds to FGF receptor 1c (FGFR1c) and its co-receptor α-klotho in the kidney. This results in (1) internalization and decreased gene expression of the sodium-dependent phosphate transporters from the Slc34 family, preventing phosphate reabsorption and in (2) inhibition of 1,25-dihydroxyvitamin D (1,25(OH)_2_D) synthesis by inhibiting the expression of 1α-hydroxylase.^30^^,^^31^^,^^32^ Lower 1,25(OH)_2_D levels will result in reduced active phosphate absorption by the intestine.^33^^,^^34^

*In vitro* and *in vivo* studies thus far looking into the direct response of FGF23 to phosphate have been inconclusive. Some controlled feeding studies in human subjects found increased serum FGF23 levels after consumption of a high phosphate containing meal, while others did not.^35^^,^^36^^,^^37^^,^^38^^,^^39^ A similar discrepancy was found *in vitro*; several studies saw no increase in *FGF23* transcripts when osteocytes were treated with inorganic phosphate,^40^^,^^41^^,^^42^ while others found elevated expression in response to inorganic phosphate treatment.^43^^,^^44^ The investigation of direct impacts of organic and inorganic phosphates in *in vivo* feeding experiments proves challenging due to the absence of information regarding the specific circulatory forms of these phosphates. However, in a recent study by Simic et al. (2020), mice were injected with either organic glycerol-3-phosphate (G-3-P) or inorganic sodium hydrogen phosphate, bypassing intestinal absorption. Only the mice injected with G-3-P had elevated FGF23 levels, indicating that osteocytes may respond differently to organic and inorganic phosphate.^45^^,^^46^

Comparative studies on organic versus inorganic phosphate mostly focus on their consequences *in vivo* and adverse health outcomes of these types of phosphate. However*, in vivo* studies often do not provide mechanistic insights at the cellular and molecular level. Studies about whether FGF23 secreting cells respond differently to these two phosphate sources are inconclusive. This paper will explore the response of FGF23-producing cells to both types of phosphate *in vitro*, to study whether their effects on FGF23 differ. To this end, murine MC3T3-E1 cells were treated with organic and inorganic phosphate sources, after which RNA sequencing was performed. Here we reveal the response to organic versus inorganic phosphate and describe novel pathways controlling FGF23 production.

## Results

### FGF23 is only regulated by organic phosphate

**Figure 1:**
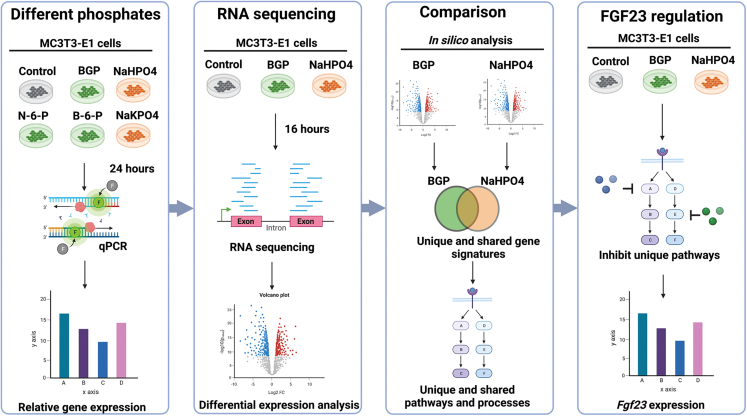
Overview of the experimental set-up Different phosphates. MC3T3-E1 cells were treated with different types of organic (green) and inorganic (phosphates) at day 27 for 24 h, after which a qPCR for *Fgf23* and *Dmp1* was performed. RNA sequencing. BGP (organic) and NaHPO4 (inorganic) were used for RNA sequencing after phosphate treatment at day 27. Comparison. Gene signatures of BGP and NaHPO4 were compared to find uniquely regulated genes and pathways by each type of phosphate. FGF23 regulation. Compounds were used to study whether found regulated genes were involved in the regulation of FGF23. Created with BioRender.com.

**Figure 2:**
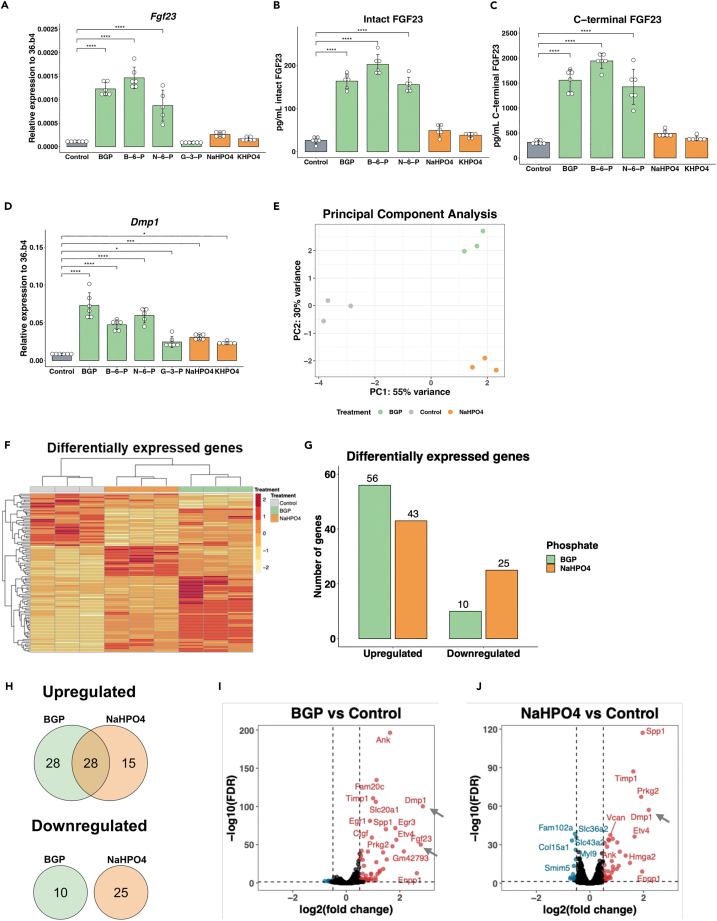
Only organic phosphate regulates FGF23 (A) Expression of *Fgf23* by organic and inorganic phosphate in which the gray bar represents the control, green bars represent organic phosphate, and orange bars represent inorganic phosphate. (B and C) Secretion of (B) C-terminal and (C) intact FGF23 after treatment with organic and inorganic phosphate in which the gray bar represents the control, green bars represent organic phosphate, and orange bars represent inorganic phosphate. (D) Expression of *Dmp1* by organic and inorganic phosphate in which the gray bar represents the control, green bars represent organic phosphate, and orange bars represent inorganic phosphate. (E) PCA plot for the following treatments: Control (gray), 4 mM BGP (green), and 4 mM NaHPO4 (orange). (F) Heatmap based on differentially expressed genes (DEGs) for the following treatments: Control (gray), 4 mM BGP (green), and 4 mM NaHPO4 (orange). (G) Total DEGs for BGP (green) and NaHPO4 (orange). (H) Venn diagrams for shared and unique DEGs by BGP (green) and NaHPO4 (orange). (I) Volcano plot DEGs by BGP treatment in which blue dots represent downregulated genes and red dots represent upregulated genes. (J) Volcano plot for DEGs regulated by NaHPO4 in which blue dots represent downregulated genes and red dots represent upregulated genes. Gene expression was normalized to housekeeping gene *36.b4*. Error bars indicate ±SEM. Significance was indicated as following: ∗*p* < 0.05, ∗∗∗*p* < 0.001, ∗∗∗∗*p* < 0.0001. Abbreviations: BGP: β-glycerophosphate, B-6-P: β-glucosamine-6-phosphate, N-6-P: N-acetyl-D-galactosamine-6 phosphate, NaHPO4: Na_2_HPO_4_/NaH_2_PO_4_, KHPO4: K_2_HPO_4_/KH_2_PO_4_.

To study the response of FGF23-producings cells to different types of phosphate, the murine mouse cell line MC3T3-E1 was differentiated into osteocyte-like cells, expressing osteocyte-markers *Dmp1*, *Fgf23*, and *Sost*, in three weeks and then cultured for 1 week in the absence of BGP (Figures S1A–S1C). Addition of dexamethasone to the cell culture medium was essential for the differentiation of the cells (Figures S1D–S1F). In order to study the different effects of organic and inorganic phosphate on FGF23-secreting cells, MC3T3-E1 cells were treated with several organic and inorganic phosphates for 24 hours followed by qPCR analysis and ELISA for FGF23. Subsequently, RNA sequencing was performed to gain more insight in transcriptional changes post-treatment. Gene signatures of organic and inorganic phosphate were compared to identify uniquely regulated genes for each type of phosphate. Finally experiments with specific compounds were performed to determine whether regulated genes are involved in the regulation of FGF23 (Figure 1). First MC3T3-E1 cells were treated with β-glycerophosphate (BGP), phosphate β-glucosamine-6-phosphate (B-6-P), N-acetyl-D-galactosamine-6 phosphate (N-6-P), or glycerol-3-phosphate (G-3-P) as organic phosphate sources or Na_2_HPO_4_/NaH_2_PO_4_ (NaHPO4) or with K_2_HPO_4_/KH_2_PO_4_ (KHPO4) as a source of inorganic phosphate for 24 h at day 27 of cell culture after which gene expression was studied using qPCR. Expression of *Fgf23* was increased by BGP (12.8-fold), B-6-P (10.9-fold), and N-6-P (9.2-fold), while treatment with G-3-P, NaHPO4, or KHPO4 failed to do so (Figure 2A). Moreover, secretion of intact FGF23 (6.2, 7.7 and 5.9-fold, respectively) and C-terminal FGF23 (5, 6.2 and 4.5-fold, respectively) was increased by BGP, B-6-P or N-6-P, but not by NaHPO4 and KHPO4 (Figures 2B and 2C), while no FGF23 was detectable in the conditioned medium from the cells treated with G-3-P (data not shown). Interestingly, another known phosphate response gene, *Dmp1*, was upregulated by all types of phosphate (Figure 2D). Taken together, we found that organic phosphates (BGP, B-6-P, and N-6-P) significantly increased *Fgf23* expression and secretion of intact and C-terminal FGF23 in differentiated MC3T3-E1 cells, while inorganic phosphates (NaHPO4 and KHPO4) did not, suggesting distinct effects of different phosphate sources on FGF23. Intriguingly, despite previous studies demonstrating upregulation of *Fgf23* in response to G-3-P treatment, G-3-P did not have this effect on the regulation of *Fgf23* in our experiments.^45^

### Treatment with BGP and NaHPO4 results in distinct gene expression profiles

To investigate how organic phosphates stimulate the expression and secretion of FGF23, we performed RNA sequencing comparing gene expression patterns after treatment with BGP as organic phosphate or NaHPO4 as inorganic phosphate. To study the spatial distribution between the samples after RNA sequencing, principal-component analysis (PCA) was performed. PCA showed that the three groups (Control, BGP, and NaHPO4) were separated into three different clusters based on two principal components explaining 85% of the variance in the dataset (Figure 2E). The heatmap of differentially expressed genes (DEGs) compared to the control indicates that all three replicates for each condition showed similar expression profiles. Moreover, it shows different clusters of up- and downregulated genes for the different treatments (Figure 2F). Together, these data indicate that FGF23-producing cells show a distinct response toward different types of phosphate. Further analyses of the DEGs showed that BGP upregulated 56 and downregulated 10 genes, while NaHPO4 upregulated 43 genes and downregulated 25 genes compared to control samples (Figure 2G, Tables S1 and S2). Twenty-eight of the upregulated but none of the downregulated genes overlapped between the two types of phosphate (Figure 2H). Moreover, the upregulated genes by BGP did not overlap with the downregulated genes by NaHPO4 and vice versa. In agreement with earlier results, *Fgf23* (indicated with arrow in Figure 2 panel I) showed a 7-fold increase in response to BGP but was not regulated by NaHPO4 (Figures 2I and 2J), while *Dmp1* (indicated with arrow in Figure 2 panel I and J) was upregulated by both BGP (2.9-fold) and NaHPO4 (2.8-fold). Moreover, these results were confirmed in the murine osteocyte cell line OmGFP66 *Fgf23* was only regulated by BGP (7.9-fold), while *Dmp1* was regulated by both BGP (99.6-fold) and NaHPO4 (39.3-fold) (Figures S1G and S1H).^47^

### ALP expression and activity in response to organic and inorganic phosphate

Once added to cell culture medium, BGP is rapidly degraded into inorganic phosphate by alkaline phosphatase (ALP, encoded by *ALPL*).^48^ To study whether BGP or NaHPO4 affects this, we measured ALP activity and expression after addition of BGP or NaHPO4 to the cell culture medium. Expression of *Alpl* was unchanged 24-h after BGP treatment, while NaHPO4 treatment decreased the expression of *Alpl* (Figure S1I). On the other hand, ALP activity was decreased by BGP and unaffected by NaHPO4 (Figure S1J). These results indicate a nuanced response of ALP to different phosphate sources, emphasizing potential complexities in the interplay between organic and inorganic phosphates and their effects on ALP-mediated processes within our experimental model. Moreover, the ALP activity rate in our model implies that BGP is converted to inorganic phosphate in this model, while directly added inorganic phosphate does not increase FGF23.

### BGP and NaHPO4 each regulate unique pathways and processes

**Figure 3:**
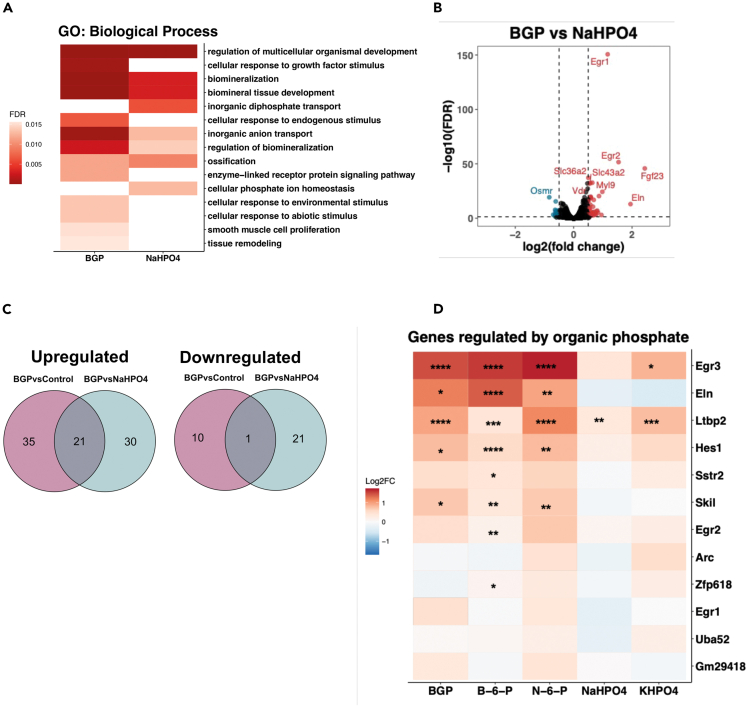
BGP and NaHPO4 regulate unique pathways and processes (A) Results from gene ontology (GO) analysis comparing the top 15 most significantly enriched biological processes for treatment with 4 mM BGP and 4 mM NaHPO4 in which the color represents the false discovery rate (FDR). (B) Volcano plot DEGs by BGP treatment versus control in which blue dots represent downregulated genes and red dots represent upregulated genes. (C) Venn diagrams of genes that are regulated in both BGP versus control (purple) and BGP versus NaHPO4 (blue) conditions. (D) Heatmap of genes regulated uniquely by BGP tested on different phosphates by qPCR, color represents the log2 fold change (Log2FC) after treatment with 4 mM of the indicated phosphate. Gene expression was normalized to housekeeping gene *36.b4*. Error bars indicate ±SEM. Significance was indicated as following: ∗*p* < 0.05, ∗∗*p* < 0.01, ∗∗∗*p* < 0.001, ∗∗∗∗*p* < 0.0001. Abbreviations: BGP: β-glycerophosphate, B-6-P: β-glucosamine-6-phosphate, N-6-P: N-acetyl-D-galactosamine-6 phosphate, NaHPO4: Na_2_HPO_4_/NaH_2_PO_4_, KHPO4: K_2_HPO_4_/KH_2_PO_4_.

Gene ontology (GO) analysis was performed to provide functional interpretation of the 56 and 44 upregulated DEGs by BGP and NaHPO4, respectively. Several GO-terms were enriched in both treatment groups, while others were uniquely enriched by either of the two phosphate treatments (Figures 3A, S2A, and S2B). Interestingly, terms indicating that cells respond to an environmental change (GO:0104004: cellular response to environmental stimulus and GO:0007167: enzyme-linked receptor protein signaling pathway) were only enriched in cells treated with BGP (Figure 3A, Table S3). GO-terms indicating regulation of cellular phosphate metabolism (GO:0005315: inorganic diphosphate transport and GO:0030643: cellular phosphate ion homeostasis) were only enriched in cells treated with NaHPO4 (Figure 3A, Table S4).

Gene set expression analysis (GSEA) was performed on the full dataset to study whether certain Hallmark gene sets were enriched following the phosphate treatments (Figure S2C). The only gene set significantly enriched by both BGP and NaHPO4 treatment was “KRAS SIGNALING UP”. In addition to KRAS signaling, BGP resulted in significant enrichment of the gene sets for the following signaling pathways: “IL2 STAT5 SIGNALING”, “TGF-β SIGNALING”, and “TNF-α SIGNALING VIA NF-κB”. Other significant enriched gene sets by organic phosphate included “ESTROGEN RESPONSE EARLY”, “EPITHELIAL MESENCHYMAL TRANSITION”, “APOPTOSIS”, and “APICAL JUNCTION”. NaHPO4 on the other hand resulted in enrichment of the gene set for “MTORC1 SIGNALING” and was enriched for several gene sets implicating involvement in cell proliferation, namely “E2F TARGETS”, “G2M CHECKPOINT”, and “MYC TARGETS V1” (Figure S2C). Furthermore, inorganic phosphate treatment led to enrichment of gene sets for “ALLOGRAFT REJECTION” and “PROTEIN SECRETION”. Together these data show that processes and signaling pathways regulated by different types of phosphate are highly distinctive.

To generate a more stringent list of genes only regulated by BGP, we compared the transcriptome following the BGP treatment with that of NaHPO4. For this, we compared the list of DEGs following BGP treatments versus the control (Figure 2J) and versus NaHPO4 treatment (Figure 3B, Tables S1 and S5). Genes that were differentially expressed after BGP treatment compared to both the control and NaHPO4 treatment were selected for further study as they show the same regulation pattern as *Fgf23*. This resulted in a list of 21 upregulated genes and 1 downregulated gene (Figure 3C). Of these 22 DEGs, 9 showed a stronger upregulation in response to BGP than to NaHPO4, while 13 genes, including *Fgf23*, were uniquely regulated by BGP (Table S6). Apart from *Fgf23* (Figure 2B), the other 12 genes were validated using the other phosphates N-6-P, B-6-P, and KHPO4 to see whether they responded similarly to BGP and NaHPO4. Of the 12 genes tested, three were significantly regulated by the organic phosphate but not by the inorganic phosphate (Figure 3D) and therefore elastin (*Eln*), hairy enhancer of split-1 (*Hes1*) and SKI-like proto-oncogene (SnoN, encoded by *Skil*) were marked as potentially associated with FGF23, as they have the same regulation pattern as *Fgf23*. Early growth response (*Egr*) −1, −2, and −3 were included in the analysis as positive controls, as they are known downstream targets of MAPK signaling, which has been associated with FGF23 regulation before.^49^^,^^50^^,^^51^

Using IPA, upstream regulator analysis was performed on all genes differentially expressed after treatment with BGP to screen for potential upstream regulators in the dataset. This revealed not only several upstream regulators including phosphate but also TGF-β1 (Figure S2D), which is central to the TGF-β signaling pathway as found in the GSEA analysis (Figure S2C).

### MAPK and TGF-β signaling are involved in regulation of FGF23 by phosphate

**Figure 4:**
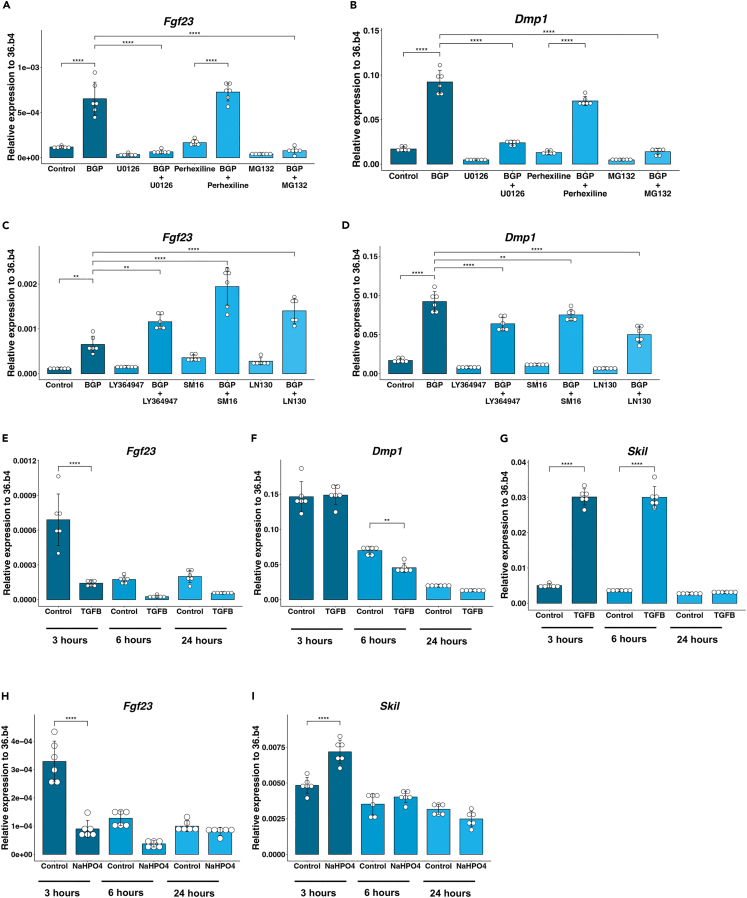
Regulation of FGF23 by different phosphate through MAPK and TFG-β signaling (A and B) Expression of (A) *Fgf23* and (B) *Dmp1* after treatment with 4 mM BGP, 10 μM MEK inhibitor U0126, HES1 inhibitor 15 μM perhexiline, proteasome inhibitor 1 μM MG132, or a combination. (C and D) Expression of (C) *Fgf23* and (D) *Dmp1* after treatment with 4 mM BGP, TGF-β inhibitors: 5 μM LY364947, 10 μM SM16 or 10 μM IN130, or a combination. (E–G) Expression of (E) *Fgf23,* (F) *Dmp1* and (G) *Skil* after addition of 10ng/mL TFG-β for 3, 6 or 24 h. (H and I) Expression of (H) *Skil* and (I) *Fgf23* after addition of 4 mM NaHPO4 for 3, 6 or 24 h. Gene expression was normalized to housekeeping gene *36.b4*. Error bars indicate ±SEM. Significance was indicated as following: ∗∗*p* < 0.01, ∗∗∗*p* < 0.001, ∗∗∗∗*p* < 0.0001.

To further examine whether EGR1, EGR2, and EGR3, HES1, SKIL, and TGF-β signaling are directly involved in the regulation of FGF23, inhibitors were used against these proteins and signaling pathways. ELN was excluded from further scrutiny as we found no possibility to inhibit it in differentiated MC3T3-E1 cells. MEK inhibitor U0126 was used to inhibit MAPK signaling.^52^ Both *Fgf23* and *Dmp1* expression were decreased when U0126 was used (Figures 4A and 4B). BGP increased *Fgf23* (F(1,20) = 55.07, *p* < 0.0001) and *Dmp1* F(1,20) = 296.14, *p* < 0.0001) while U0126 decreased the expression of *Fgf23* F(df) = 77.78, *p* < 0.0001) and *Dmp1* F(df) = 215.42, *p* < 0.0001). The combined treatment of U0126 and BGP resulted in significantly lower expression levels of both *Fgf23* and *Dmp1* compared to the treatment with BGP alone (Figures 4A and 4B). Two-way ANOVA analyses showed a strong significant interaction effect between BGP and U0126 in regulation of both *Fgf23* (F(1,20) = 55.07, *p* < 0.0001) and *Dmp1* (F(1,20) = 296.14, *p* < 0.0001), implicating the functional involvement of MAPK signaling in regulation of FGF23 and DMP1 expression by BGP. The response to BGP by *Egr1*, *-2*, and -*3* was inhibited by MEK inhibitor U0126, but no significant interaction between BGP and U0126 was found, while *Hes1*, *Skil*, and *Eln* were unaffected by U0126 (Figures S3A–S3F). Together these data indicate that expression of *Fgf23*, *Dmp1*, *Egr1*, *2*, and *3* are regulated by the MAPK signaling pathway.

Perhexiline, a HES1 gene expression signature antagonist,^53^ did not inhibit BGP-induced regulation of *Fgf23* or *Dmp1* expression, indicating that it is unlikely that HES1 is a regulator of FGF23 (Figures 4A and 4B). The other genes of interest were also not affected by perhexiline, suggesting that HES1 does not have a regulating role, but rather is co-regulated with FGF23.

Skil (SnoN), a protein that exhibited the same regulation pattern as FGF23 in our earlier analyses, is known for its negative regulation of TGF-β signaling—a pathway identified as regulated by BGP in our GSEA analysis—is degraded by the ubiquitin-proteasome pathway. The proteasome inhibitor MG132 has been shown to stabilize SnoN and decrease TGF-β activation.^54^ Interestingly, MG132 inhibited the expression of *Fgf23* (F(df) = 71.58, *p* < 0.0001) and *Dmp1* (F(df) = 265.07, *p* < 0.0001), indicating that SnoN may act as a negative regulator of FGF23, despite showing a similar regulatory pattern as *Fgf23* in our RNA sequencing experiment. (Figures 4A and 4B). Moreover, we observed a significant interaction effect between BGP and MG132 on both *Fgf23* (F(1,20) = 41.61, *p* < 0.0001) and *Dmp1* (F(1,20) = 140.91, *p* < 0.0001). Combining the treatments resulted in reduced *Fgf23* and *Dmp1* expression compared to BGP alone (Figures 4A and 4B). Interestingly, *Smad7*, which like SnoN is a negative regulator of TGF-β signaling, was not affected by BGP alone, but showed increased expression when BGP was combined with MEK inhibitor U0126 (Figure S3G).^55^ Together, these data indicate that stabilization of SnoN results in decreased expression of *Fgf23*, *Dmp1*, *Hes1*, *Skil*, *Eln*, *Egr1*, -*2*, and -*3*, indicating that these genes might be regulated by TGF-β. Moreover, the regulation of *Smad7* by BGP in the absence of MAPK signaling indicated that there is crosstalk between MAPK signaling and TGF-β signaling in the presence of BGP.

As MG132 is non-specific for SnoN, we performed additional experiments to show that indeed TGF-β signaling is involved in the regulation of FGF23. To this end, three different inhibitors of the TGF-β signaling pathway were used. LY364947 inhibits both canonical and non-canonical signaling by blocking phosphorylation of TGF-β receptors 1 and 2, SM16 inhibits phosphorylation of SMAD2 and SMAD3, while IN130 inhibits SMAD2 phosphorylation and its nuclear translocation.^56^ LY364947 (F(1, 20) = 22.26, *p* = 0.0001), SM16 (F(1, 20) = 29.75, *p* = 0.0001), and IN130 (F(1,20) = 17.58, *p* = 0.0004) all synergistically increased *Fgf23* expression (Figure 4C). In contrast, the *Dmp1* response to BGP was decreased in the presence of each of the TGF-β inhibitors, with significant interaction between LY364947 (F(1,20) = 9.46, *p* = 0.006) or IN130 (F(1,20) = 22.13, *p* = 0.0001) and BGP (Figure 4D). The response of *Hes1*, *Eln*, and *Egr3* was not altered by TGF-β inhibitors, while expression of *Skil* was decreased regardless of BGP (Figures S4A–S4C and S4F). The expression of *Egr1* and -*2* was slightly increased by BGP when SMAD phosphorylation was inhibited by SM16 but remained unchanged following treatment with either of the other two inhibitors (Figures S4D and S4E). Smad7 was downregulated by LY364947 and IN130 but not by SM16 (Figure S4G).

In agreement with these results, treatment with recombinant TGF-β1 resulted in decreased *Fgf23* expression within 3 h (Figure 4E), while expression of *Dmp1* was unaffected at that time point. Meanwhile, *Skil* expression was strongly increased within 3 h of TGF-β1 addition (Figure 4G). These findings suggest that TGF-β signaling exerts a negative regulatory effect on *Fgf23* expression, potentially mediated through the modulation of *Skil*. To investigate whether activation of TGF-β signaling is involved in the lack of regulation of *Fgf23* by inorganic phosphate, we studied the expression of *Skil* and *Fgf23* after 3-, 6-, and 24-h treatment with NaHPO4. Notably, we observed an upregulation of *Skil* expression 3 h after NaHPO4 treatment and decrease in *Fgf23* expression (Figures 4H and 4J).

## Discussion

Phosphate is present in most food sources either as naturally occurring organic phosphate or as added inorganic phosphate.^2^ Previous studies have shown that inorganic dietary phosphate increases circulating phosphate and FGF23 levels, while organic phosphate does not have this effect.^23^^,^^24^ The current study was performed to determine the effect of both types of phosphate on murine FGF23-producing cells. It showed that treatment of MC3T3-E1 osteocyte-like cells with the organic phosphates BGP, B-6-P, or N-6-P resulted in higher expression and secretion of FGF23, while the inorganic phosphates NaHPO4 and KHPO4 did not affect FGF23, these results were validated by RNA sequencing. Interestingly, G-3-P, a kidney-derived organic phosphate previously shown to increase FGF23 levels when directly injected into mice, did not affect *Fgf23* expression in our study.^45^ Gene ontology analysis showed that processes enriched in BGP-treated cells were associated with a cell responding to an environmental change, while cells treated with NaHPO4 were enriched for processes involved in maintaining ion homeostasis, indicating that organic and inorganic phosphate have very distinct effects on osteocyte-like cells. Finally, we found that FGF23 is regulated by organic phosphate through a balance of both MAPK and TGF-β signaling. The transporter by which organic phosphate can enter the cell or the receptor to which it can bind is thus far unknown. It had been speculated that phosphate transporters PiT1 (SLC20A1) and PiT1 (SLC20A2) or FGFR1c function as phosphate sensors. These could also be the potential sensors of organic phosphate.^57^ However, more research into how organic phosphate can affect these pathways is needed.

### Effects of phosphate on FGF23 expression

Studies on the direct effect of phosphate on FGF23 production and secretion have not been conclusive thus far. Our study might provide an explanation for these discrepancies, as the source of phosphate seems to be essential for its ability to stimulate FGF23. Several controlled feeding studies in humans have shown that high phosphate intake resulted in higher levels of FGF23, while lower intake resulted in a decrease.^35^^,^^36^^,^^37^ Some of these studies also reported higher serum phosphate levels when subjects were fed a meal with high inorganic phosphate, while others did not.^38^^,^^39^ Moreover, glial cells missing homolog2 (*Gcm2*) null mice which suffered from hyperphosphatemia, and MC3T3-E1 cultures treated with phosphate did not have elevated *Fgf23* transcripts.^40^^,^^41^^,^^58^ A study by Takashi et al. showed increased serum FGF23 levels in mice fed a high phosphate diet, but after further studies in UMR106 cells, they hypothesized that this was due to decreased FGF23 cleavage rather than increased FGF23 production.^42^ Nonetheless, some studies have shown that addition of phosphate *in vitro* does result in increased FGF23 expression.^43^^,^^44^ Additionally, the study by Simic et al. showed that organic phosphate G-3-P increased serum FGF23 levels while sodium hydrogen phosphate did not. In our study, G-3-P did not stimulate the expression of *Fgf23*. It is noteworthy that, in contrast to our study, Simic et al. did not demonstrate direct effects of G-3-P on FGF23 in an *in vitro* cell culture system. This suggests that G-3-P may undergo additional modifications or the presence of intermediate molecules *in vivo,* which could be essential for its stimulatory effect on FGF23. The discrepancy in the response of G-3-P between our study and the work by Simic et al. highlights the need for further investigation of the complex interactions between phosphate and FGF23.^45^

### Role for ALP in FGF23 stimulation

While ALP was not the focus of this paper, its potential role in FGF23 activation by BGP is interesting. Previous studies have shown that when BGP is added to culture medium, it is rapidly converted to inorganic phosphate.^48^ Beck et al. (2000) have shown that generation of free phosphate by ALP after addition of BGP is necessary for the induction of osteopontin (OPN) expression.^59^ However, in this study, addition of inorganic phosphate also stimulated the expression of *Opn*, while in our study inorganic phosphate failed to stimulate *Fgf23*. It is therefore unlikely that the inorganic phosphate resulting from the conversion of BGP stimulates FGF23. However, since BGP had a lowering effect on ALP activity, while NaHPO4 did not, we cannot exclude that ALP is involved in the regulation of FGF23 by BGP. Future research should take this potential role of ALP into account by performing experiments both in the presence and absence of ALP.

### Organic phosphate stimulates FGF23 through MAPK and TGF-β signaling

The fact that only organic phosphate regulated FGF23 in this study, while DMP1 was regulated by both types of phosphate thereby indicating that both types of phosphate elicited a different phosphate response, provided us with the opportunity to get a deeper understanding of FGF23 regulation. As described before by Takashi et al., inhibition of MAPK signaling regulates the FGF23 response of osteocytes to phosphate via EGR1 and -2. However, they stated that this was due to increased expression of *GalNt3*, which prevents FGF23 cleavage.^42^ Here we show that organic phosphate can directly stimulate FGF23 and DMP1 through MAPK signaling. Inhibition of MAPK signaling inhibited both the response of *Fgf23* and *Dmp1* to BGP as well as *Egr1*, *2*, and *3*, but not *Hes1*, *Skil*, and *Eln*. Therefore, it is likely that *Fgf23* is directly regulated by MAPK signaling through *Egr1*, *2*, and *3*.

Interestingly, MG132 which has been described to inhibit the degradation of SnoN, encoded by *Skil*, also inhibited the response of *Fgf23* to BGP.^60^ This was unexpected as *Skil* followed the expression pattern of *Fgf23* in response to organic phosphate. It has been described previously that *Skil* expression is negatively regulated by the SMAD/SnoN transcriptional repressor complex.^61^ To date, very few other genes have been described as a target for the SMAD/SnoN complex.^62^ Our data indicates that *Skil* might play a role in repressing *Fgf23* expression suggesting a complex regulatory role of the SMAD/SnoN transcriptional repressor complex in the modulation of *Fgf23* and controlling the response to phosphate. Inhibition of SMAD2/3 phosphorylation and nuclear translocation resulted in increased *Fgf23* expression in the absence of BGP, while the addition of recombinant TGF-β1 resulted in less *Fgf23* transcripts. Moreover, inhibition of TGF-β signaling resulted in decreased expression of *Skil*. Together, this indicates that TGF-β1 is a negative regulator of FGF23 and that upregulation of *Skil* could possibly control this signaling pathway in response to BGP.^56^ Of note, TGF-β2 has been described to have a stimulating effect on FGF23 in UMR106 cells, indicating different forms of TGF-β might have opposite effects on FGF23.^63^ Together, our results indicate that *Fgf23* is regulated by both MAPK signaling and TGF-β signaling, whereby disturbances in either of these pathways result in deregulated *Fgf23* expression. Finally, we found that NaHPO4 quickly upregulates expression of *Skil* while downregulating *Fgf23*. These findings suggest a potential link between *Skil* and the absence of FGF23 activation in response to inorganic phosphate. However, it is important to exercise caution and emphasize that additional research is needed to fully elucidate the underlying mechanisms and confirm this relationship.

### Potential implications of differential effects of phosphate types on FGF23

To our knowledge, we are the first to compare the direct effects of different types of phosphate at the level of FGF23 synthesizing cells. Typically, when studying the effects of phosphate interventions, cells or animals are treated with NaHPO4, which, based on our current findings, would explain the lack of FGF23 stimulation found in some of these studies. Moreover, our study has important implications as to how we should interpret the relationship between dietary phosphate and FGF23. Based on our findings, there is an urgent need to investigate how increased consumption of inorganic phosphate over the last decades may have influenced the organic/inorganic phosphate ratio in our circulation and what that entails at the level of the osteocytes. Our findings indicate that when this ratio is shifted toward inorganic phosphate, this may result in insufficient phosphate clearance and disturbed homeostasis consequently. It is also consistent with observations in several epidemiological studies describing that elevated or even high-normal serum (inorganic) phosphate levels are associated with the risk of cardiovascular events, fracture risk, and premature aging.^2^^,^^14^^,^^26^^,^^64^ Understanding the precise mechanisms by which different forms of phosphate impact on FGF23 expression and secretion may have implications for the development of therapeutic strategies targeting phosphate metabolism related disorders and associated bone disorders but may also lead to nutritional interventions to reduced inorganic phosphate intake.

### Limitations of the study

As our findings were generated *in vitro* and were conducted in two murine osteocyte-like cell lines, they cannot be directly translated to *in vivo* effects of a high dietary inorganic phosphate intake or other *in vitro* models. Therefore, validation of these results in different models is essential toward gaining understanding in the regulation of FGF23 by both organic and inorganic phosphates. Additionally, an important consideration in our study is the consistent presence of 1 mM sodium phosphate monobasic (NaH_2_PO4-H_2_O) in the alpha-MEM formulation across all conditions, which may have introduced a potential confounding factor. Future study designs should carefully evaluate the impact of this ambient phosphate concentration on FGF23 regulation. To date, there is little knowledge on how phosphate is processed after intestinal absorption and the ratio of serum organic/inorganic phosphate is barely studied. It is therefore currently impossible to implicate a correlation between dietary inorganic and inorganic serum phosphate levels and an inappropriate FGF23 response. However, the fact that serum phosphate levels only rise with a high inorganic phosphate diet in animal studies indicates that the FGF23 being synthesized is not sufficient to normalize phosphate homeostasis.^23^^,^^24^ Additionally, our study did not consider the role of parathyroid hormone (PTH). Recent research by Centeno et al. (2019) has highlighted the potential involvement of the calcium sensing receptor in the parathyroid as a phosphate sensor. Inorganic phosphate has been shown to stimulate the secretion of PTH, which, in turn, can enhance the secretion of FGF23 by osteocytes.^65^ To shed light on these complex regulatory systems, it is important that future research focuses on controlled feeding studies comparing high inorganic to organic phosphate dietary loads.

### Conclusion

In conclusion, our findings provide valuable molecular and mechanistic insights into the response of FGF23-producing cells to either inorganic or organic phosphate. Specifically, we have demonstrated that FGF23, a key regulator of phosphate homeostasis, is solely influenced by organic phosphate. Speculatively, if the use of inorganic phosphates as food additives were to increase the ratio of inorganic phosphate in serum, it is plausible that this shift could potentially lead to adverse health effects. Therefore, it is crucial to study how our changing diets alter the circulating inorganic/organic phosphate ratio in our bodies, since too much inorganic phosphate may disturb phosphate homeostasis.

## STAR★Methods

### Key resources table

**Table:** 

REAGENT or RESOURCE	SOURCE	IDENTIFIER
**Chemicals, peptides, and recombinant proteins**		

Alpha-minimum essential medium (αMEM)	Gibco	Cat#: A10490-01
Fetal bovine serum (FBS)	Gibco	Cat#: F7524-500ML Batch#: 0001661160
Beta-glycerophosphate	Sigma-Aldrich	Cat#: G5422-100G
Ascorbic acid	Sigma-Aldrich	Cat#: A4403
Dexamethasone	Sigma-Aldrich	Cat#: A4544
Rat tail collagen type 2	Sigma-Aldrich	Cat#: D4902
β-glucosamine-6-phosphate (B-6-P)	Sigma-Aldrich	Cat#: G5509-10MG
N-acetyl-D-galactosamine-6 phosphate (N-6-P)	Sigma-Aldrich	Cat#: 92689-10MG
Glycerol-3-phosphate (G3P)	Sigma-Aldrich	Cat#: G7886-1G
U0126	MedChemExpress	Cat#: HY-12031A
LY364947	MedChemExpress	Cat#: HY-13462
SM16	MedChemExpress	Cat#: HY-111482
IN130	MedChemExpress	Cat#: HY-18758
Perhexiline	Sigma-Aldrich	Cat#: SML0120
MG132	MedChemExpress	Cat#: HY-13259
TRIzol reagent	ThermoFisher Scientific	Cat#: 15596018

**Critical commercial assays**		

C-terminal FGF23 mouse/rat ELISA	Immutopics	Cat#: 60-6300
Intact FGF23 mouse/rat ELISA	Immutopics	Cat#: 60-6800
RevertAid First Strand cDNA Synthesis Kit	ThermoFisher Scientific	Cat#: K1622
Truseq RNA stranded polyA library prep kit	Illumina	Cat#: 20020595
Agilent RNA 6000 Nano Kit	Agilent	Cat#: 5067-1511

**Deposited data**		

RAW data	This paper	GSE245758

**Experimental models: Cell lines**		

MC3T3-E1 clone#4	ATCC	Cat#: CRL2593
OmGFP66	Kind gift from Sarah Dallas	N/A

**Software and algorithms**		

R studio	Posit PBC	
Artificial Intelligence RNA-seq (AIR)	Sequentia Biotech	
Ingenuity Pathway Analysis	Qiagen	

### Resource availability

#### Lead contact

Further information and requests for resources and reagents should be directed to and will be fulfilled by the lead contact, Bram van der Eerden (b.vandereerden@erasmusmc.nl).

#### Materials availability

This study did not generate new unique reagents.

#### Data and code availability

• RNA-seq data have been deposited at GEO and is publicly available as of the date of publication. The accession number is listed in the key resources table.
• This paper does not report original code.
• Any additional information required to reanalyze the data reported in this paper is available from the lead contact upon request.

### Experimental model and study participant details

#### Cells

MC3T3-E1 subclone #4 were obtained from ATCC (Virginia, USA) and OmGFP66 were gifted by Sarah Dallas. Both MC3T3-E1 were kept in alpha-minimum essential medium (αMEM; A10490-01, Gibco, Paisley, UK), supplemented with 10% fetal bovine serum (Gibco), 100 Units/mL penicillin and 100 μg/mL streptomycin (Gibco) in a humid environment containing 5% CO2 at 37°. OmGFP66 were kept in the same medium as MC3T3-E1 cells supplemented with 2 mM L-glutamine (Gibco) in a humid environment containing 5% CO2 at 37°.

### Method details

#### Cell culture

The murine pre-osteoblastic MC3T3-E1 cell line was cultured and passaged in proliferation medium, αMEM supplemented with 10% fetal bovine serum, 100 Units/mL penicillin and 100 μg/mL streptomycin. For experiments, 2.0∗10^4^ cells were seeded in 12-wells plates and kept for 2 days in proliferation medium before being switched to osteogenic medium (αMEM, 10% FBS, 100 Units/mL penicillin and 100 μg/mL streptomycin, 10 mM β-glycerophosphate (BGP, Sigma-Aldrich, Missouri, United States), 50 μg/mL ascorbic acid (Sigma-Aldrich) and 0.1 μM dexamethasone (Sigma-Aldrich).^66^ The osteocyte cell line OmGFP66 was cultured and passaged in proliferation medium. For experiments, 7.0∗10^4^ were seeded in a 12-wells plate coated with 0.15 mg/mL rat tail collagen type 2 (Sigma-Aldrich) and kept in proliferation medium until confluency was reached. Cells were then switched to osteogenic medium (αMEM, 3% FBS, 100 Units/mL penicillin and 100 μg/mL streptomycin, 5 mM β-glycerophosphate, 50 μg/mL ascorbic acid and 2 mM L-glutamine.^47^ Cells were differentiated into FGF23-producing cells for 21 days, after which β-glycerophosphate was removed from the differentiation medium and cultures were continued until day 28. Cells were treated for 24 h on day 27, unless indicated otherwise, before being lysed to obtain total RNA as described below. Expression of osteocyte markers at day 28 was assessed by quantitative real-time PCR.

For RNA sequencing, cells were treated with either 4 mM BGP or a mixture of Na_2_HPO_4_ and NaH_2_PO_4_ (NaHPO4, pH 7.4, Sigma-Aldrich) for 16 h. For validation purposes, cells were treated with 4 mM of 5 different types of phosphate: BGP, NaHPO4, K_2_HPO_4_/KH_2_PO_4_ (KHPO4, pH 7.4, Sigma-Aldrich), β-glucosamine-6-phosphate (B-6-P, Sigma-Aldrich) and N-acetyl-D-galactosamine-6 phosphate (N-6-P, Sigma-Aldrich), glycerol-3-phosphate (G3P, Sigma-Aldrich). For other experiments, cells were treated with 4 mM BGP or 4 mM NaHPO4 combined with either 10 μM MEK inhibitor U0126, TGF-β inhibitors LY364947 (5 μM; MedChemExpress, New Jersey, USA), SM16 (10 μM; MedChemExpress), IN130 (10 μM; MedChemExpress), HES1 inhibitor perhexiline (15 μM; Sigma-Aldrich), proteasome inhibitor MG132 (1 μM; Sigma-Aldrich), human recombinant TGF-β1 (10 ng/mL; Cedarlane, Burlington, Canada) or any of these compounds alone. 1 mM sodium phosphate monobasic (NaH2PO4-H2O) was consistently present in all experimental conditions, as part of the αMEM formulation. Figure 1 summarizes the phosphates and compounds that were used for each type of experiment.

#### RNA isolation, cDNA synthesis and quantitative real-time PCR

Cells were lysed in TRIzol Reagent (ThermoFisher Scientific, Massachusetts, USA), and 1/5 volume of chloroform was added for phase separation. Samples were centrifuged at 14,000 x *g* for 20 min and the aqueous phase was collected. RNA was precipitated by adding an equal volume of isopropanol to the aqueous phase followed by overnight incubation at −20°C. The next day, samples were centrifuged 30 min at 14,000 x *g* at 4°C. The supernatant was discarded, and samples were washed with 100% ethanol, followed by an incubation with 0.1 M EDTA (Invitrogen, Massachusetts, USA) and 8 M Lithium Chloride (Merck, New Jersey, USA) overnight at −20°C to remove hydroxyapatite and other minerals present in the extracellular matrix. Then, samples were centrifuged for 30 min at 14,000 x *g* and 4°C and washed three times with 70% ethanol, followed by a final wash in 100% ethanol. Finally, pellets were dissolved in RNase-free H_2_O. Total RNA concentration was determined using the NanoDrop 8000 Spectrophotometer (ThermoFisher Scientific, Massachusetts, USA). One μg of total RNA was reverse transcribed using the RevertAid First Strand cDNA Synthesis Kit (ThermoFisher Scientific) according to the manufacturer’s protocol. Gene expression was evaluated by quantitative real-time PCR using a QuantStudio 7 Flex Real-Time PCR system (Applied Biosystems, Massachusetts, USA) and SYBR green PCR master mix reagent (Promega, Wisconsin, USA). All primer sets were designed to span at least one exon-exon junction (Table S7). In order to calculate the relative expression of the genes of interest, the Ct values of the target genes were subtracted from the housekeeping gene *36.b4* in order to obtain the ΔCt. and expressed as 2^−ΔCt^.

#### Enzyme-linked ImmunoSorbent assay (ELISA)

Conditioned medium was collected from cells and directly stored at −80°C. To determine Intact and C-terminal Fgf23, the ELISA kits Mouse/Rat Fgf-23 (Intact) and Mouse/Rat Fgf-23 (C-Term) (both from Immutopics, California, USA) were used according to the manufacturer’s protocols.

#### Alkaline phosphatase and protein assays

Alkaline phosphatase (ALP) and protein measurements were performed at day 28 of cell culture as described previously.^67^ ALP activity was determined by an enzymatic reaction, where the ALP-mediated conversion of *para*-nitrophenylphosphate (pNPP) (Sigma) to paranitrophenol (PNP). Thawed cellular extracts were sonicated using a Soniprep 150 (Sanyo) until homogeneous. 10 μL sample was mixed with 90 μL PBS/Triton (0.1%) (Sigma-Aldrich). To each tube, 100 μL of *p*-NPP was added, vortexed, and incubated in a water bath at 37°C. After 10 min, the reaction was stopped by adding 250 μL of 0.1 M sodium hydroxide (Sigma-Aldrich). Absorbance was measured at 405 nm using a microplate reader. ALP results were adjusted for protein content of the cell lysates. To this end Pierce BCA Protein Assay Kit (ThermoFisher Scientific) was used. Fresh WR was prepared by mixing 50 parts of BCA reagent A with 1 part of BCA reagent B (50:1, Reagent A to B). 25 μL of each standard or unknown sample replicate was pipetted into a microplate well. Following this, 200 μL of the WR was added to each well and the plate was thoroughly mixed. The plate incubated at 37°C for 30 min in the dark.). The absorbance at 595 nm was measured using a plate reader.

#### RNA sequencing and bioinformatics analysis

RNA isolation was performed as described above. RNA concentration and size distributions were analyzed on an Agilent Bioanalyzer RNA 6000 Nano chip (ThermoFisher Scientific). RNA sequencing library was prepared using the Truseq RNA stranded polyA library prep kit (Illumina, California, United States). Sequencing was performed at 2 × 50 bp on a NovaSeq 6000 (Illumina). To analyze RNA-seq data the Artificial Intelligence RNA-seq (AIR) was used (https://sequentiabiotech.com/). RNA-seq data validation, pairing and FastQC quality control were performed after which the reads were mapped against the mouse (Genbank: GRCm39/Ensembl, release 104) genome. For identification of differentially expressed genes (DEGs) the EdgeR method was used within AIR. Genes with a log2 fold-change >0.5 and a false discovery rate (FDR) < 0.05 were considered significantly different.

Gene Ontology analysis was done using the R package clusterProfiler (version 4.6.0),^68^ and GO terms were trimmed using REVIGO (https://revigo.irb.hr). Gene set enrichment analysis (GSEA) was performed on the normalized TPM values using Hallmark analysis within GSEA (version 4.2.3, Broad Institute). Ingenuity Pathway Analysis (IPA; Ingenuity Systems) was used to predict target pathways and study interactions between overrepresented genes.

### Quantification and statistical analysis

The data are shown as mean ± standard error of mean (SEM), in which n represents the number of individual samples within an experiment. Two groups were compared using an unpaired Student’s t test. In case of more than 2 groups, a one-way analysis of variance (ANOVA) followed by the Tukey post-hoc test was performed. When combinations of treatments were used, a two-way ANOVA was performed to study the interactions between the compounds. The results of the two-way ANOVA were reported as F(df) = F-value, *p*-value, where F(df) represents the F-value with corresponding degrees of freedom and *p*-value indicates the statistical significance of the analysis. Differences were considered significant if *p* < 0.05. Significance was indicated as following: ∗*p* < 0.05, ∗∗*p* < 0.01, ∗∗∗*p* < 0.001, ∗∗∗∗*p* < 0.0001. All statistical analyses were done using R studio rstatix package (version 0.7.1).

## Supplemental information

Document S1. Figures S1–S4, Tables S6 and S7

Table S1. Differentially expressed genes in MC3T3-E1 treated with 4 mM β-glycerophosphate for 16 h, related to Figure 2

Table S2. Differentially expressed genes in MC3T3-E1 treated with 4 mM Na_2_HPO_4_/NaH_2_PO_4_, related to Figure 2

Table S3. Enriched GO terms (Biological Process) for MC3T3-E1 treated with 4 mM β-glycerophosphate for 16 h, related to Figure 3

Table S4. Enriched GO terms (Biological Process) for MC3T3-E1 treated with 4 mM Na_2_HPO_4_/NaH_2_PO_4_, related to Figure 3

Table S5. Differentially expressed genes in MC3T3-E1 for 4 mM β-glycerophosphate versus 4 mM Na_2_HPO_4_/NaH_2_PO_4_, related to Figure 3

## References

[1] Cooke A. (2017). Dietary food-additive phosphate and human health outcomes. Compr. Rev. Food Sci. Food Saf..

[2] Ritz E., Hahn K., Ketteler M., Kuhlmann M.K., Mann J. (2012). Phosphate additives in food—a health risk. Dtsch. Arztebl. Int..

[3] Sullivan C.M., Leon J.B., Sehgal A.R. (2007). Phosphorus-containing food additives and the accuracy of nutrient databases: implications for renal patients. J. Ren. Nutr..

[4] Erem S., Razzaque M.S. (2018). Dietary phosphate toxicity: an emerging global health concern. Histochem. Cell Biol..

[5] Cupisti A., Kalantar-Zadeh K. (2013).

[6] Mathewson A.M., Fouque D., Toft A.J. (2010). Dietary phosphate assessment in dialysis patients. J. Ren. Nutr..

[7] Sabbagh Y., Giral H., Caldas Y., Levi M., Schiavi S.C. (2011). Intestinal phosphate transport. Adv. Chron. Kidney Dis..

[8] Levi M., Gratton E., Forster I.C., Hernando N., Wagner C.A., Biber J., Sorribas V., Murer H. (2019). Mechanisms of phosphate transport. Nat. Rev. Nephrol..

[9] Bell R.R., Draper H.H., Tzeng D.Y., Shin H.K., Schmidt G.R. (1977). Physiological responses of human adults to foods containing phosphate additives. J. Nutr..

[10] Peacock M. (2021). Phosphate metabolism in health and disease. Calcif. Tissue Int..

[11] Jain N., Elsayed E.F. (2013). Dietary phosphate: what do we know about its toxicity. J. Nephrol..

[12] Miyamoto K.-i., Oh J., Razzaque M.S. (2022). Phosphate Metabolism.

[13] Chang A.R., Lazo M., Appel L.J., Gutiérrez O.M., Grams M.E. (2014). High dietary phosphorus intake is associated with all-cause mortality: results from NHANES III. Am. J. Clin. Nutr..

[14] Kuro-o M. (2010). A potential link between phosphate and aging—lessons from Klotho-deficient mice. Mech. Ageing Dev..

[15] Chung L.-H., Liu S.-T., Huang S.-M., Salter D.M., Lee H.-S., Hsu Y.-J. (2020). High phosphate induces skeletal muscle atrophy and suppresses myogenic differentiation by increasing oxidative stress and activating Nrf2 signaling. Aging (Albany NY).

[16] Lau W.L., Linnes M., Chu E.Y., Foster B.L., Bartley B.A., Somerman M.J., Giachelli C.M. (2013). High phosphate feeding promotes mineral and bone abnormalities in mice with chronic kidney disease. Nephrol. Dial. Transplant..

[17] Richter B., Kapanadze T., Weingärtner N., Walter S., Vogt I., Grund A., Schmitz J., Bräsen J.H., Limbourg F.P., Haffner D., Leifheit-Nestler M. (2022). High phosphate-induced progressive proximal tubular injury is associated with the activation of Stat3/Kim-1 signaling pathway and macrophage recruitment. FASEB J..

[18] Jin H., Hwang S.-K., Yu K., Anderson H.K., Lee Y.-S., Lee K.H., Prats A.-C., Morello D., Beck G.R., Cho M.-H. (2006). A high inorganic phosphate diet perturbs brain growth, alters Akt-ERK signaling, and results in changes in cap-dependent translation. Toxicol. Sci..

[19] Lee S., Kim J.-E., Hong S.-H., Lee A.-Y., Park E.-J., Seo H.W., Chae C., Doble P., Bishop D., Cho M.-H. (2015). High inorganic phosphate intake promotes tumorigenesis at early stages in a mouse model of lung cancer. PLoS One.

[20] Jin H., Xu C.-X., Lim H.-T., Park S.-J., Shin J.-Y., Chung Y.-S., Park S.-C., Chang S.-H., Youn H.-J., Lee K.-H. (2009). High dietary inorganic phosphate increases lung tumorigenesis and alters Akt signaling. Am. J. Respir. Crit. Care Med..

[21] Ugrica M., Gehring N., Giesbertz P., Pastor-Arroyo E.M., Daniel H., Wagner C.A., Rubio-Aliaga I. (2022). Chronic High Phosphate Intake in Mice Affects Macronutrient Utilization and Body Composition. Mol. Nutr. Food Res..

[22] Kawamura H., Tanaka S., Ota Y., Endo S., Tani M., Ishitani M., Sakaue M., Ito M. (2018). Dietary intake of inorganic phosphorus has a stronger influence on vascular-endothelium function than organic phosphorus. J. Clin. Biochem. Nutr..

[23] Coltherd J.C., Staunton R., Colyer A., Thomas G., Gilham M., Logan D.W., Butterwick R., Watson P. (2019). Not all forms of dietary phosphorus are equal: an evaluation of postprandial phosphorus concentrations in the plasma of the cat. Br. J. Nutr..

[24] Dobenecker B., Reese S., Herbst S. (2021). Effects of dietary phosphates from organic and inorganic sources on parameters of phosphorus homeostasis in healthy adult dogs. PLoS One.

[25] Cozzolino M., Foque D., Ciceri P., Galassi A. (2017). Phosphate in chronic kidney disease progression. Contrib. Nephrol..

[26] Foley R.N., Collins A.J., Herzog C.A., Ishani A., Kalra P.A. (2009). Serum phosphorus levels associate with coronary atherosclerosis in young adults. J. Am. Soc. Nephrol..

[27] Campos-Obando N., Bosman A., Kavousi M., Medina-Gomez C., van der Eerden B.C.J., Bos D., Franco O.H., Uitterlinden A.G., Zillikens M.C. (2022). Genetic evidence for a causal role of serum phosphate in coronary artery calcification: the rotterdam study. J. Am. Heart Assoc..

[28] Erben R.G., Andrukhova O. (2017). FGF23-Klotho signaling axis in the kidney. Bone.

[29] Ubaidus S., Li M., Sultana S., De Freitas P.H.L., Oda K., Maeda T., Takagi R., Amizuka N. (2009). FGF23 is mainly synthesized by osteocytes in the regularly distributed osteocytic lacunar canalicular system established after physiological bone remodeling. J. Electron. Microsc..

[30] Quarles L.D. (2012). Skeletal secretion of FGF-23 regulates phosphate and vitamin D metabolism. Nat. Rev. Endocrinol..

[31] Brunette M.G., Chan M., Ferriere C., Roberts K.D. (1978). Site of 1, 25 (OH) 2 vitamin D3 synthesis in the kidney. Nature.

[32] Verstuyf A., Carmeliet G., Bouillon R., Mathieu C. (2010). Vitamin D: a pleiotropic hormone. Kidney Int..

[33] Hernando N., Pastor-Arroyo E.M., Marks J., Schnitzbauer U., Knöpfel T., Bürki M., Bettoni C., Wagner C.A. (2021). 1, 25 (OH) 2 vitamin D3 stimulates active phosphate transport but not paracellular phosphate absorption in mouse intestine. J. Physiol..

[34] Wilz D.R., Gray R.W., Dominguez J.H., Lemann J. (1979). Plasma 1, 25-(OH) 2-vitamin D concentrations and net intestinal calcium, phosphate, and magnesium absorption in humans. Am. J. Clin. Nutr..

[35] Ferrari S.L., Bonjour J.-P., Rizzoli R. (2005). Fibroblast growth factor-23 relationship to dietary phosphate and renal phosphate handling in healthy young men. J. Clin. Endocrinol. Metab..

[36] Antoniucci D.M., Yamashita T., Portale A.A. (2006). Dietary phosphorus regulates serum fibroblast growth factor-23 concentrations in healthy men. J. Clin. Endocrinol. Metab..

[37] Gutiérrez O.M., Wolf M., Taylor E.N. (2011). Fibroblast growth factor 23, cardiovascular disease risk factors, and phosphorus intake in the health professionals follow-up study. Clin. J. Am. Soc. Nephrol..

[38] Nishida Y., Taketani Y., Yamanaka-Okumura H., Imamura F., Taniguchi A., Sato T., Shuto E., Nashiki K., Arai H., Yamamoto H., Takeda E. (2006). Acute effect of oral phosphate loading on serum fibroblast growth factor 23 levels in healthy men. Kidney Int..

[39] Ito N., Fukumoto S., Takeuchi Y., Takeda S., Suzuki H., Yamashita T., Fujita T. (2007). Effect of acute changes of serum phosphate on fibroblast growth factor (FGF) 23 levels in humans. J. Bone Miner. Metabol..

[40] Bon N., Frangi G., Sourice S., Guicheux J., Beck-Cormier S., Beck L. (2018). Phosphate-dependent FGF23 secretion is modulated by PiT2/Slc20a2. Mol. Metabol..

[41] Camalier C.E., Yi M., Yu L.R., Hood B.L., Conrads K.A., Lee Y.J., Lin Y., Garneys L.M., Bouloux G.F., Young M.R. (2013). An integrated understanding of the physiological response to elevated extracellular phosphate. J. Cell. Physiol..

[42] Takashi Y., Kosako H., Sawatsubashi S., Kinoshita Y., Ito N., Tsoumpra M.K., Nangaku M., Abe M., Matsuhisa M., Kato S. (2019). Activation of unliganded FGF receptor by extracellular phosphate potentiates proteolytic protection of FGF23 by its O-glycosylation. Proc. Natl. Acad. Sci. USA.

[43] Hori M., Kinoshita Y., Taguchi M., Fukumoto S. (2016). Phosphate enhances Fgf23 expression through reactive oxygen species in UMR-106 cells. J. Bone Miner. Metabol..

[44] Ito N., Findlay D.M., Anderson P.H., Bonewald L.F., Atkins G.J. (2013). Extracellular phosphate modulates the effect of 1α, 25-dihydroxy vitamin D3 (1, 25D) on osteocyte like cells. J. Steroid Biochem. Mol. Biol..

[45] Simic P., Kim W., Zhou W., Pierce K.A., Chang W., Sykes D.B., Aziz N.B., Elmariah S., Ngo D., Pajevic P.D. (2020). Glycerol-3-phosphate is an FGF23 regulator derived from the injured kidney. J. Clin. Invest..

[46] Zhou W., Simic P., Zhou I.Y., Caravan P., Vela Parada X., Wen D., Washington O.L., Shvedova M., Pierce K.A., Clish C.B. (2023). Kidney glycolysis serves as a mammalian phosphate sensor that maintains phosphate homeostasis. J. Clin. Invest..

[47] Wang K., Le L., Chun B.M., Tiede-Lewis L.M., Shiflett L.A., Prideaux M., Campos R.S., Veno P.A., Xie Y., Dusevich V. (2019). A novel osteogenic cell line that differentiates into GFP-tagged osteocytes and forms mineral with a bone-like lacunocanalicular structure. J. Bone Miner. Res..

[48] Bellows C.G., Heersche J.N., Aubin J.E. (1992). Inorganic phosphate added exogenously or released from β-glycerophosphate initiates mineralization of osteoid nodules in vitro. Bone Miner..

[49] He Q., Shumate L.T., Matthias J., Aydin C., Wein M.N., Spatz J.M., Goetz R., Mohammadi M., Plagge A., Divieti Pajevic P., Bastepe M. (2019). AG protein–coupled, IP3/protein kinase C pathway controlling the synthesis of phosphaturic hormone FGF23. JCI insight.

[50] Yamazaki M., Ozono K., Okada T., Tachikawa K., Kondou H., Ohata Y., Michigami T. (2010). Both FGF23 and extracellular phosphate activate Raf/MEK/ERK pathway via FGF receptors in HEK293 cells. J. Cell. Biochem..

[51] Ewendt F., Föller M. (2019). p38MAPK controls fibroblast growth factor 23 (FGF23) synthesis in UMR106-osteoblast-like cells and in IDG-SW3 osteocytes. J. Endocrinol. Invest..

[52] Favata M.F., Horiuchi K.Y., Manos E.J., Daulerio A.J., Stradley D.A., Feeser W.S., Van Dyk D.E., Pitts W.J., Earl R.A., Hobbs F. (1998). Identification of a novel inhibitor of mitogen-activated protein kinase kinase. J. Biol. Chem..

[53] Schnell S.A., Ambesi-Impiombato A., Sanchez-Martin M., Belver L., Xu L., Qin Y., Kageyama R., Ferrando A.A. (2015). Therapeutic targeting of HES1 transcriptional programs in T-ALL. Blood.

[54] Luo D.D., Phillips A., Fraser D. (2010). Bone morphogenetic protein-7 inhibits proximal tubular epithelial cell Smad3 signaling via increased SnoN expression. Am. J. Pathol..

[55] de Ceuninck van Capelle C., Spit M., Ten Dijke P. (2020). Current perspectives on inhibitory SMAD7 in health and disease. Crit. Rev. Biochem. Mol. Biol..

[56] Khoshakhlagh M., Soleimani A., Binabaj M.M., Avan A., Ferns G.A., Khazaei M., Hassanian S.M. (2019). Therapeutic potential of pharmacological TGF-β signaling pathway inhibitors in the pathogenesis of breast cancer. Biochem. Pharmacol..

[57] Beck L., Beck-Cormier S. (2020). Extracellular phosphate sensing in mammals: what do we know?. J. Mol. Endocrinol..

[58] Liu S., Tang W., Fang J., Ren J., Li H., Xiao Z., Quarles L.D. (2009). Novel regulators of Fgf23 expression and mineralization in Hyp bone. Mol. Endocrinol..

[59] Beck G.R., Zerler B., Moran E. (2000). Phosphate is a specific signal for induction of osteopontin gene expression. Proc. Natl. Acad. Sci. USA.

[60] Smith D.M., Patel S., Raffoul F., Haller E., Mills G.B., Nanjundan M. (2010). Arsenic trioxide induces a beclin-1-independent autophagic pathway via modulation of SnoN/SkiL expression in ovarian carcinoma cells. Cell Death Differ..

[61] Tecalco-Cruz A.C., Sosa-Garrocho M., Vázquez-Victorio G., Ortiz-García L., Domínguez-Hüttinger E., Macías-Silva M. (2012). Transforming growth factor-β/SMAD Target gene SKIL is negatively regulated by the transcriptional cofactor complex SNON-SMAD4. J. Biol. Chem..

[62] Tecalco-Cruz A.C., Ríos-López D.G., Vázquez-Victorio G., Rosales-Alvarez R.E., Macías-Silva M. (2018). Transcriptional cofactors Ski and SnoN are major regulators of the TGF-β/Smad signaling pathway in health and disease. Signal Transduct. Targeted Ther..

[63] Feger M., Hase P., Zhang B., Hirche F., Glosse P., Lang F., Föller M. (2017). The production of fibroblast growth factor 23 is controlled by TGF-β2. Sci. Rep..

[64] Campos-Obando N., Koek W.N.H., Hooker E.R., van der Eerden B.C., Pols H.A., Hofman A., van Leeuwen J.P., Uitterlinden A.G., Nielson C.M., Zillikens M.C. (2017). Serum phosphate is associated with fracture risk: the Rotterdam Study and MrOS. J. Bone Miner. Res..

[65] Centeno P.P., Herberger A., Mun H.-C., Tu C., Nemeth E.F., Chang W., Conigrave A.D., Ward D.T. (2019). Phosphate acts directly on the calcium-sensing receptor to stimulate parathyroid hormone secretion. Nat. Commun..

[66] Yashiro M., Ohya M., Mima T., Nakashima Y., Kawakami K., Yamamoto S., Kobayashi S., Yano T., Tanaka Y., Sonou T. (2020). Active vitamin D and vitamin D analogs stimulate fibroblast growth factor 23 production in osteocyte-like cells via the vitamin D receptor. J. Pharm. Biomed. Anal..

[67] Bruedigam C., Driel M.v., Koedam M., Peppel J.v.d., van der Eerden B.C.J., Eijken M., van Leeuwen J.P.T.M. (2011). Basic techniques in human mesenchymal stem cell cultures: differentiation into osteogenic and adipogenic lineages, genetic perturbations, and phenotypic analyses. Curr. Protoc. Stem Cell Biol..

[68] Yu G., Wang L.-G., Han Y., He Q.-Y. (2012). clusterProfiler: an R package for comparing biological themes among gene clusters. OMICS A J. Integr. Biol..

