# Supplementary material for: Organic phosphate but not inorganic phosphate regulates Fgf23 expression through MAPK and TGF-ꞵ signaling
Source: iScience. 2024 Mar 29;27(6):109625. doi: 10.1016/j.isci.2024.109625 (PMC11178987; doi:10.1016/j.isci.2024.109625)
Supplement: Document S1. Figures S1–S4, Tables S6 and S7 [file mmc1.pdf]

iScience, Volume *27*

Supplemental information

Organic phosphate but not inorganic phosphate regulates *Fgf23* expression through MAPK and TGF-β signaling

Danielle M.A. Ratsma, Max Muller, Marijke Koedam, Johannes P.T.M. van Leeuwen, M. Carola Zillikens, and Bram C.J. van der Eerden

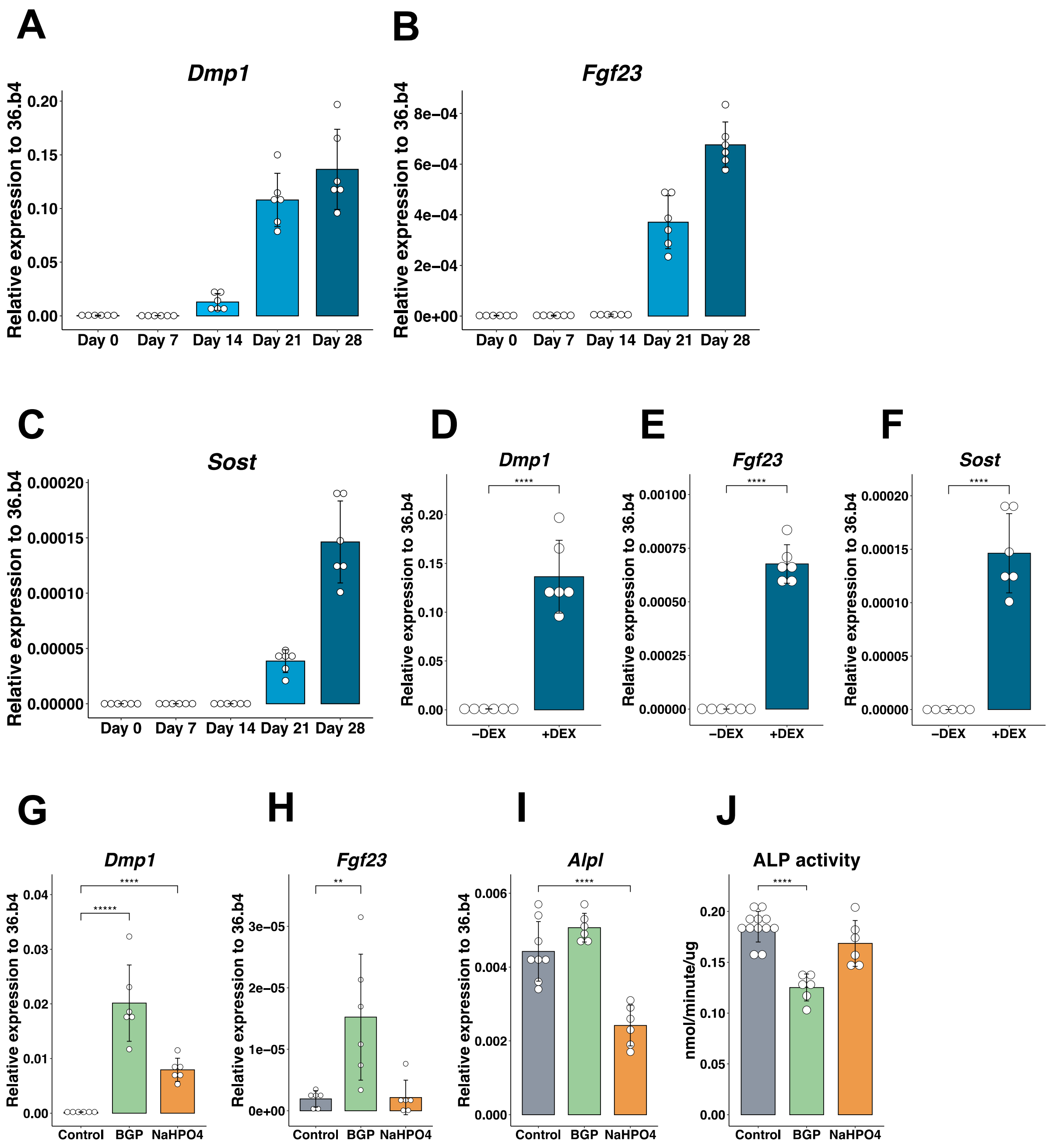

Supplementary figure 1. Effects of BGP and NaHPO4 on MC3T3-E1 and OmGFP66. (A-C) Expression of (A) Dmp1, (B) Fgf23 and (C) Sost after 28 days of osteogenic differentiation of MC3T3-E1 cells. Expression of (D) Dmp1, (E) Fgf23 and (F) Sost in MC3T3-E1 cells at day 28 after differentiation in the presence or absence of dexamethasone (DEX).

Expression of (G) *Dmp1* and (H) *Fgf23* after 24 hours treatment with BGP and NaHPO4 on day 28. (I-H) Expression of (I) *Alpl* and (J) activity of ALP (correct for protein) after 24 hours treatment with BGP and NaHPO4 on day 28. Gene expression was normalized to housekeeping gene *36.b4*. Error bars indicate ± SEM. Significance was indicated as following: ** $p < 0.01$ and **** $p < 0.0001$ (Related to figure 2).

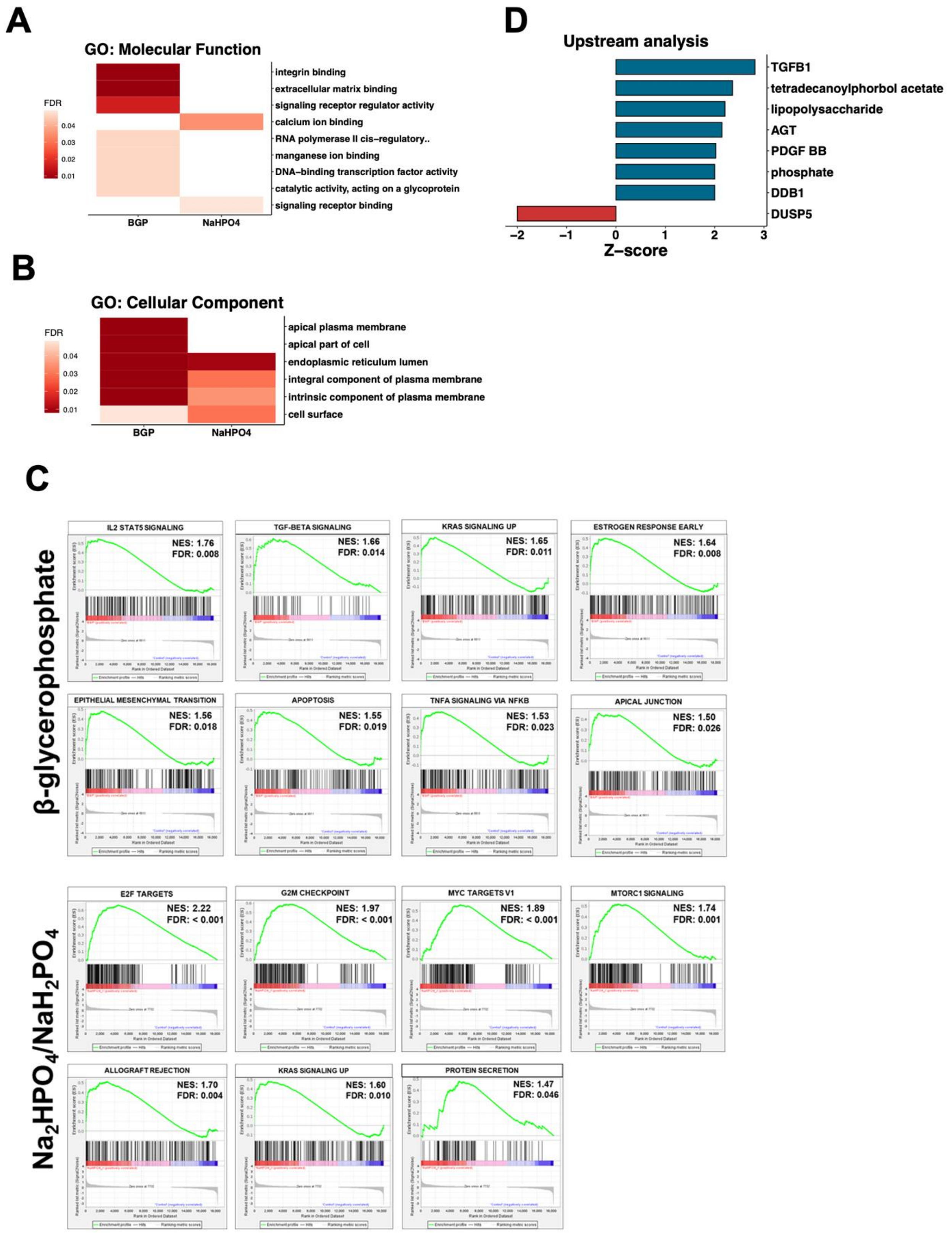

Supplementary figure 2. BGP and NaHPO4 regulate unique processes. (A-B) Results from Gene Ontology (GO) analyses comparing enriched terms for (A) molecular function and (B) cellular component for treatment with 4 mM BGP and 4 mM NaHPO4 in which the color represents the false discovery rate (FDR). (C) Results from gene set enrichment analysis (GSEA) for treatment with BGP and NaHPO4, indicating the normalized enrichment score (NES) and FDR. (D) IPA upstream regulator analysis for BGP in which blue indicates activated and red indicates inhibited (Related to figure 3).

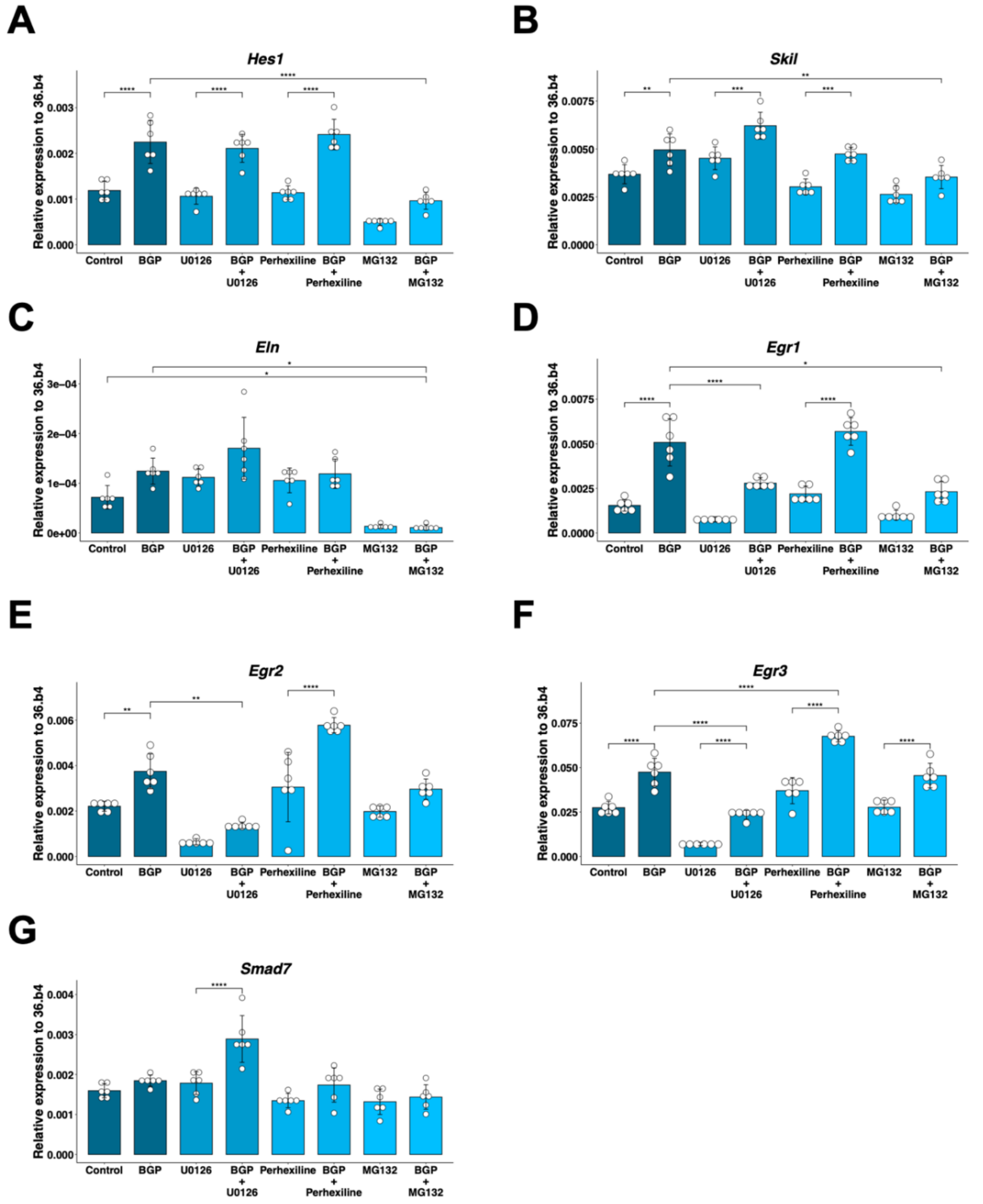

Supplementary figure 3. Regulation of genes of interest by MAPK, HES1 and SnoN. (A-F) Expression of (A) *Hes1*, (B) *Skil,* (C) *Eln,* (D) *Egr1*, (E) *Egr2*, (F) *Egr3* and (G) *Smad7* after treatment with 4 mM BGP, 10 μM MEK inhibitor U0126, HES1 inhibitor 15 μM perhexiline, proteasome inhibitor 1 μM MG132 or a combination. Gene expression was normalized to housekeeping gene *36.b4*. Error bars indicate ± SEM. Significance was indicated as following: * p < 0.05, ** p < 0.01, *** p < 0.001, **** p < 0.0001 (Related to figure 4).

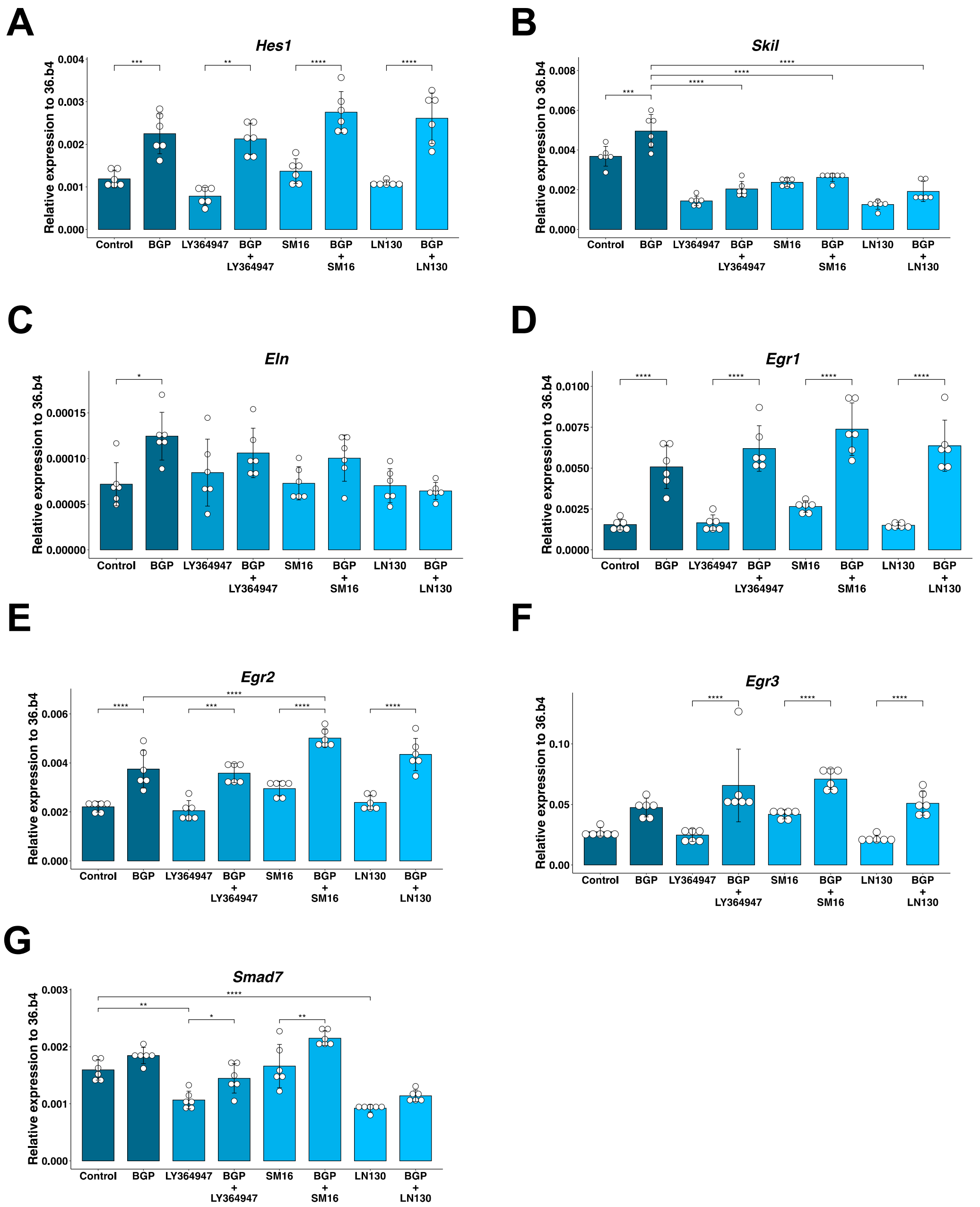

Supplementary figure 4. Regulation of genes of interest by TGF-β signaling. (A-F) Expression of (A) *Hes1*, (B) *Skil,* (C) *Eln,* (D) *Egr1*, (E) *Egr2*, (F) *Egr3,* (G) *Smad7* after treatment with 4 mM BGP, TGF-β inhibitors: 5 μM LY364947, 10 μM SM16 or 10 μM LN130 or a combination. Gene expression was normalized to housekeeping gene *36.b4*. Error bars indicate ± SEM. Significance was indicated as following: * $p < 0.05$, ** $p < 0.01$, *** $p < 0.001$, **** $p < 0.0001$ (Related to figure 4).

Gene	Log2FC BGPvsControl	Log2FC BGPvsNaHPO4	FC BGPvsControl	FC BGPvsNaHPO4
Ank	1.633404	0.522396	3.102443	1.436338
Dmp1	2.85713	0.662954	7.245725	1.583321
Egr1	**0.884152**	**1.164798**	**1.845679**	**2.242018**
Egr3	**1.831913**	**1.537771**	**3.560087**	**2.903456**
Fgf23	**2.779861**	**2.436965**	**6.86786**	**5.415014**
Gm42793	2.149324	0.659466	4.4362	1.579498
Egr2	**1.372987**	**1.537771**	**2.590064**	**2.903456**
Skil	**0.658414**	**0.590881**	**1.578346**	**1.506166**
Il12a	1.376563	0.713137	2.59649	1.639365
Trib1	1.252072	0.809666	2.381833	1.752805
Enpp1	2.635214	0.688667	6.212672	1.611793
Hivep3	0.930129	0.542285	1.905446	1.456277
Scml4	1.090464	0.722217	2.129425	1.649716
Arc	**0.937925**	**0.833078**	**1.915771**	**1.781482**
Hes1	**0.694342**	**0.659899**	**1.618147**	**1.579972**
Gm29418	**0.868227**	**0.738249**	**1.825418**	**1.668149**
Ltbp2	**0.585047**	**0.503449**	**1.500088**	**1.417598**
Sstr2	**0.793432**	**0.676541**	**1.733193**	**1.598303**
Syt12	1.104118	0.515971	2.149675	1.429957
Eln	**1.002244**	**1.947115**	**2.003114**	**3.856026**
Zfp618	**0.557792**	**0.595556**	**1.472015**	**1.511055**
Uba52	**-0.55643**	**-0.54711**	**0.679985**	**0.684389**

Supplementary table 6. Genes showing increased expression for β-glycerophosphate versus control and β-glycerophosphate versus 4 mM Na_2HPO_4/NaH_2PO_4. Genes that were considered uniquely regulated by β-glycerophosphate and not more strongly regulated by β-glycerophosphate than by Na_2HPO_4/NaH_2PO_4 are written in bold (Related to figure 3).

Gene	Forward	Reverse
36.b4	TTGGCCAATAAGGTGCCAGC	GGAGGTCTTCTCGGGTCCTA
Alpl	ACACTCGGCCGATCGGGACT	CGCCACCCATGATCACGTCGA
Arc	AGTCTTGGGCAGCATAGCTC	TGAATCACTGCTGGGGGC
Dmp1	TGTGGGAAAAAGACCTTGGGAG	GTATCTGGCAACTGGGAGAGCA
Egr1	CACCTGACCACAGAGTCCTTT	CGGCCAGTATAGGTGATGGG
Egr2	CTCGTCGGTGACCATCTTCC	TTGATCATGCCATCTCCCGC
Egr3	GCCTGACAATCTGTACCCCGAG	GGGAGGCTGTAGCCATCTGATT
Eln	GGATTGGAGGCATTGCAGGC	CACTAAACCTCCAGCAGCTCCATA
Fgf23	CCATCAGACCATCTACAGTGCC	CTTCGAGTCATGGCTCCTGTT
Hes1	AAAATTCCTCCTCCCCGGT	ATGATAGGCTTTGATGACTTTCTG
Ltbp2	CTGGCTACATTTGCCTGTGC	GCATTCGTTGTGATCCTGGC
Skil	CGGCGGGCACAGATCAATTA	ACTGAAGACTCCACCTGAACT
Sstr2	TGCTCGAGGAAAACCAAGATGT	TCCGTCTAGAACCAAGCTGC
Uba52	AGACTAGGGGGTTTCCGGTT	ATGTTGGCGTCTGCCGCT
Zfp618	CGATGCACCAGCCTCTTACA	AGGTGTTTTCTGCTGCTGCT
Gm29418	CGTGGGAACACCACTACCTC	GCTCTCAGGGCAGACCTAAC

Supplementary table 7. Primer sets used for qPCR
